# Free mate choice enhances conservation breeding in the endangered giant panda

**DOI:** 10.1038/ncomms10125

**Published:** 2015-12-15

**Authors:** Meghan S. Martin-Wintle, David Shepherdson, Guiquan Zhang, Hemin Zhang, Desheng Li, Xiaoping Zhou, Rengui Li, Ronald R. Swaisgood

**Affiliations:** 1 Division of Applied Animal Ecology, Institute for Conservation Research, San Diego Zoo Global, 15600 San Pasqual Valley Road, Escondido, California 92027, USA; 2 PDXWildlife, 9233 SW Brier Pl., Portland, Oregon 97219, USA; 3 Biology Department, Portland State University, 1719 SW 10th Avenue, SRTC rm 246, Portland, Oregon 97201, USA; 4 Conservation Division, Oregon Zoo, 4001 SW Canyon Road, Portland, Oregon 97221-9704, USA; 5 China Conservation and Research Center for the Giant Panda, Wolong, Sichuan 623006, PR China

## Abstract

Conservation breeding programmes have become an increasingly important tool to save endangered species, yet despite the allocation of significant resources, efforts to create self-sustaining populations have met with limited success. The iconic giant panda (*Ailuropoda melanoleuca*) embodies the struggles associated with *ex situ* species conservation. Here we show that behavioural mate preferences in giant pandas predict reproductive outcomes. Giant pandas paired with preferred partners have significantly higher copulation and birth rates. Reproductive rates increase further when both partners show mutual preference for one another. If managers were to incorporate mate preferences more fully into breeding management, the production of giant panda offspring for China's reintroduction programme might be greatly expedited. When extended to the increasing numbers of species dependent on *ex situ* conservation breeding to avoid extinction, our findings highlight that mate preference and other aspects of informed behavioural management could make the difference between success and failure of these programmes.

## Abstract (web summary)

*Ex situ* conservation breeding programmes for endangered species are expensive, and not always guaranteed to succeed. Here, Martin-Wintle *et al.* show that when captive giant pandas are given the opportunity to choose their preferred mate, copulation and birth rates increase significantly.

## Graphical abstract

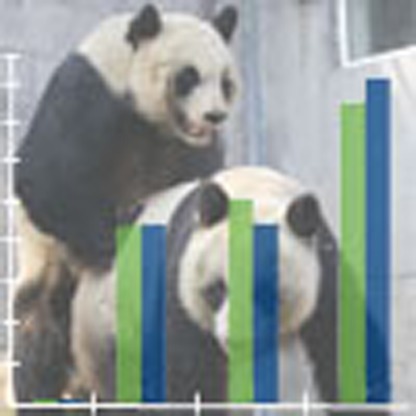

There are 7,678 vertebrate species currently listed in the International Union for Conservation of Nature (IUCN) Red List of Threatened Species^1^, with effects on ecosystem function comparable to or greater than those mediated by other global change drivers^2^. These declining species may represent incalculable loss to humanity in terms of ecosystem services alone^3^. In a recently published study using the International Species Information System database researchers found that captive breeding played a major role in the recovery efforts of 15% of the species whose threat level has been reduced^4^. As wild animal populations continue to decline at alarming rates^5^,^6^, conservation breeding is becoming an increasingly important tool to guard against extinction, providing organisms for reintroduction to re-establish or supplement wild populations, or for assisted colonization outside of historical ranges as part of climate change mitigation strategies^7^. Key to the success of reintroduction programmes is the availability of a large number of founding individuals to guarantee population establishment and maintenance of genetic diversity^8^,^9^. Thus, a primary goal of conservation breeding programmes should be to generate large numbers of genetically diverse candidates for assurance and reintroduction.

However, conservation breeding programmes are expensive and their value for conservation depends to some extent on how rapidly they can achieve high rates of population growth cost-effectively. The value of many programmes is diminished by the fact that the majority are not breeding animals to replacement (that is, where recruitment equals or exceeds mortality) and fall below the threshold of 200 individuals deemed necessary for genetically healthy and sustainable populations^10^,^11^. An important contributing factor explaining this failure may stem from the methods currently employed by conservation breeding programmes where animals are typically paired to minimize inbreeding and maintain founder representation. Many zoo and breeding facility programmes give animals a single option for a mate, precluding mate choice^12^, and the success or failure of reproduction hangs on the outcome of that one pairing. Mate incompatibility can impede captive breeding programmes^13^ by reducing reproductive rates and excluding potential genetic contributions to the population. It is therefore surprising that mate preferences have not figured more prominently in captive breeding programmes.

Mate preference has been recognized as an important factor in evolutionary ecology since Darwin posed it as a mechanism of sexual selection^14^,^15^. It has been shown to affect copulation success^16^, offspring production^16^,^17^ and offspring survivorship^16^,^17^,^18^. Animals mated to their preferred partner have increased reproductive performance in insects^19^, fish^20^, birds^21^,^22^ and mammals^17^,^18^. Fitness benefits resulting from female mate preference are most common, but male mate preference may also have fitness consequences^18^. Because females tend to have lower potential reproductive output than males and invest more heavily in offspring, females are expected to evolve more selective mating preferences. However, contrary to prevailing wisdom, which characterizes females as choosy and males as indiscriminate, male mate choice may also be prevalent in species without paternal care where large investment in mating effort by males limits the number of females that can be inseminated^23^. Failure to address mutual mate preferences by both males and females may be misleading, and recent reviews have identified research in this area as one of the most pressing needs for obtaining a better understanding of sexual selection^14^,^23^. In mammals, stringent tests of mutual mate preference are rare, although mutual mate preference may be common place^24^, and its importance has not yet been evaluated in conservation breeding situations. It is easy to imagine how evaluating mate preference in conservation breeding programmes, where it is traditionally underemphasized, could potentially increase reproductive performance in endangered species.

Here we report the effect of male, female and mutual mate preference on reproductive performance in a captive bred population of giant pandas. In addition to preference, we examine multiple husbandry and physiological factors that may be contributing to differential reproductive performance and report the factors that contribute most to successful mate pairings.

Here we investigate the effects of female, male and mutual mate preference on reproductive outcomes using dichotomous choice tests in the giant panda. We find breeding female and male giant pandas to a preferred mate increases intromission success from as low as 0% where both partners are non-preferred to as much as 80% where both partners are preferred. In addition, factoring out copulation behaviour, mate preferences further contributed to an increase in offspring production. These findings suggest that mate preference should be incorporated into current captive breeding management to maximize reproductive success in these programmes. We discuss implications for *ex situ* conservation breeding programmes.

## Results

### Female reproductive performance

**Figure 1:**
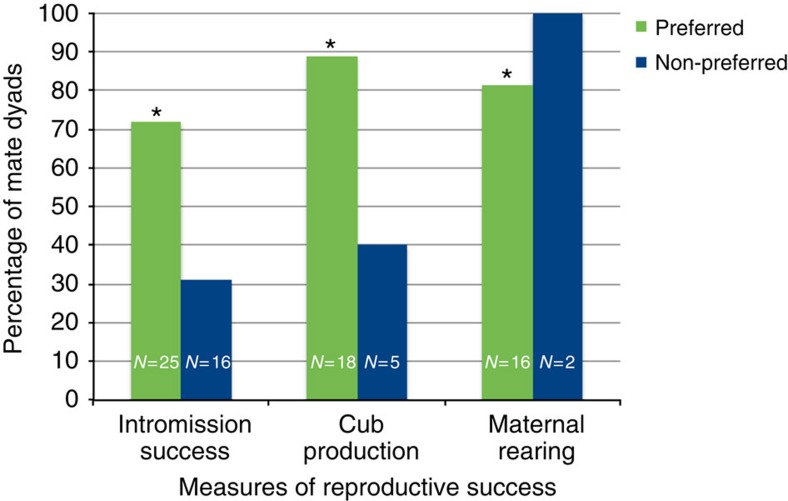
Female giant panda preference. Data have been pooled across years and total sample sizes are shown on the columns. Green bars represent preferred mates and blue bars represent non-preferred mates. Intromission success (χ^2^=7.35, *P*=0.007); cub production (χ^2^=10.9, *P*=0.001); maternal rearing^†^ (χ^2^=8.07, *P*=0.005). *indicates *P*≤0.05 for *χ*^2^ test. *P* values were obtained via χ^2^ tests for graphing purposes only. ^†^Low sample sizes preclude statistical analysis in a GLMM for this variable.

This experiment compared reproductive performance of female giant pandas mated to their preferred male with females mated to their non-preferred male, as determined via behavioural scoring before mate introductions in dichotomous choice tests. Using generalized linear mixed models (GLMM; SPSS 19.0 for Mac OS X) with studbook ID as a random effect, we found an overall significance of preference of female giant pandas that were mated to their preferred mate having more successful intromissions (GLMM, *B*=2.63, Wald *χ*^2^_1,41_=155.21, *P*<0.001, Bayesian Information Criterion (BIC)=175.26; *χ*^2^-test, χ^2^=7.35, *P*=0.007) and producing more cubs (*B*=4.91, *Wald χ*^2^_1,41_=11.35, *P*=0.001, BIC=31.48; *χ*^2^-test, χ^2^=10.9, *P*=0.001) than females mated to their non-preferred mate (Supplementary Table 1 and 2). Sample sizes for our maternal rearing variable for non-preferred males dropped below those required for statistical analysis in a logistic regression as only two cubs were maternally reared by this group. Female reproductive performance was notably higher when mated with preferred than non-preferred partners (Table 1, Fig. 1, Supplementary Table 1 and 2). Intromission was more than twice as likely with preferred males and among those that copulated females were approximately two times more likely to produce a cub with a preferred male.

We investigated several additional explanatory variables in our GLMM logistic regressions, including measures of familiarity between the male and female, weight, age, provenance (captive- versus wild-born), rearing and mating history (see Methods). Most of these explanatory variables showed no relationship with the reproductive performance by female giant pandas, however, we found a significant effect of previous litter production (*B*=0.65, *Wald χ*^2^_1,41_=0.03, *P*<0.001, BIC=175.26) and male mass (B=0.08, *Wald χ*^2^_1,41_=3.02, *P*=0.003, BIC=175.26) on intromission success (Supplementary Table 1). There was also a significant effect of male age (*B*=−0.46, *Wald χ*^2^_1,41_=6.93, *P*=0.008, BIC=31.48), male weight (*B*=−0.17, *Wald χ*^2^_1,41_=12.28, *P*<0.001, BIC=31.48), and female rearing (*B*=−3.74, *Wald χ*^2^_1,41_=5.48, *P*<0.019) on cub production (Supplementary Table 2). Parent reared females were significantly more likely to produce a cub (81% of successful intromissions) than hand reared females (66% of successful intromissions). In addition, females were more likely to breed successfully and produce cubs with older, larger males (mean male age=13.14, mean male weight=123.9 kg) than younger smaller males (mean male age=11.5, mean male weight=104.8 kg).

### Male reproductive performance

This experiment compared reproductive performance of male giant pandas mated to their preferred female with males mated to their non-preferred female. GLMMs for male reproductive success measurements included the same response and explanatory variables listed above for females; however, the explanatory factor ‘focal animal rearing' was removed from our final model for male reproductive performance because all males in our data set were mother reared.

**Figure 2:**
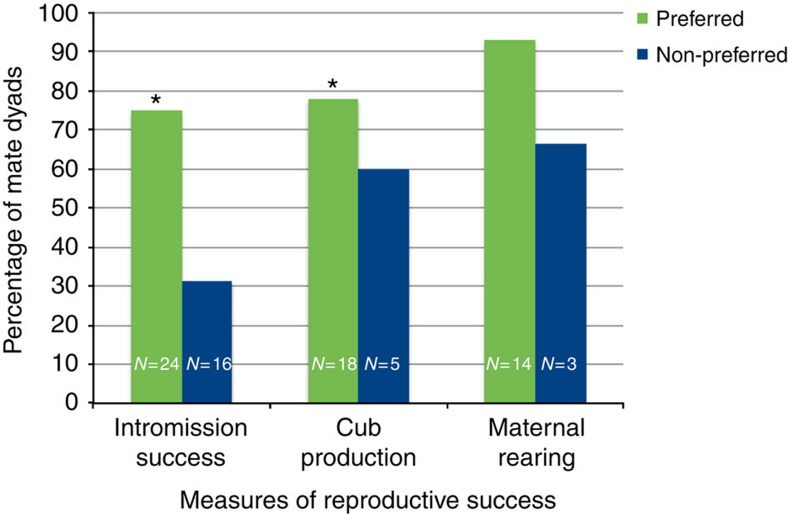
Male giant panda preference. Data have been pooled across years and total sample sizes are shown on the columns. Green bars represent preferred mates and blue bars represent non-preferred mates. Intromission success (χ^2^=7.35, *P*=0.007); cub production (χ^2^=7.12, *P*=0.008); maternal rearing† (χ^2^=8.07, *P*=0.005). *indicates *P*≤0.05 for *χ*^2^ test. *P* values were obtained via χ^2^ tests for graphing purposes only. ^†^Low sample sizes preclude statistical analysis in a GLMM for this variable.

Using GLMM (SPSS 19.0 for Mac OS X) with studbook ID as a random effect, we found males mated to their preferred mate have more successful intromissions (GLMM, *B*=1.44, *Wald χ*^2^_1,40_=2.70, *P*=0.100, BIC=49.73; *χ*^2^-test, χ^2^=7.35, *P*=0.007) and produced more cubs (GLMM, B=1.64, *Wald χ*^2^_1,40_=2.32, *P*=0.128, BIC=42.01; *χ*^2^-test, χ^2^=7.12, *P*=0.008) than males mated to their non-preferred mate (Supplementary Table 3 and 4). See the Methods section for a discussion of current accepted practice for reporting of all factors included in the final best-fit model, regardless of *P* value^25^. Sample sizes for our maternal rearing variable for non-preferred females dropped below those required for statistical analysis in logistic regression. Similar to female preference, male mate preference had a large effect on reproductive measurements (Table 2, Fig. 2), with males achieving intromission more than twice as frequently with preferred females than with non-preferred females. For males that did copulate, those mating with preferred females were more likely to produce cubs than those mating with non-preferred females. In addition, older males had higher rates of intromission and cub production.

Most of the other explanatory variables showed no relationship with the reproductive performance by male giant pandas. However, we found an effect of previous litter production (*B*=2.01, *Wald χ*^2^_1,40_=3.32, *P*=0.069, BIC=49.73) on intromission success (Supplementary Table 3) and there was also a significant effect of male mass (*B*=−0.58, *Wald χ*^2^_1,40_=5.90, *P*=0.015, BIC=42.01) on cub production (Supplementary Table 4). Males paired to females they had previously sired cubs with were more likely to have successful intromission (90% success) than males paired with females that they had not sired their cubs (43% success). Larger males (mean male weight=125.1 kg) were more likely to produce cubs than smaller males (mean male weight=110.2 kg)

### Mutual mate preference and reproductive performance

**Figure 3:**
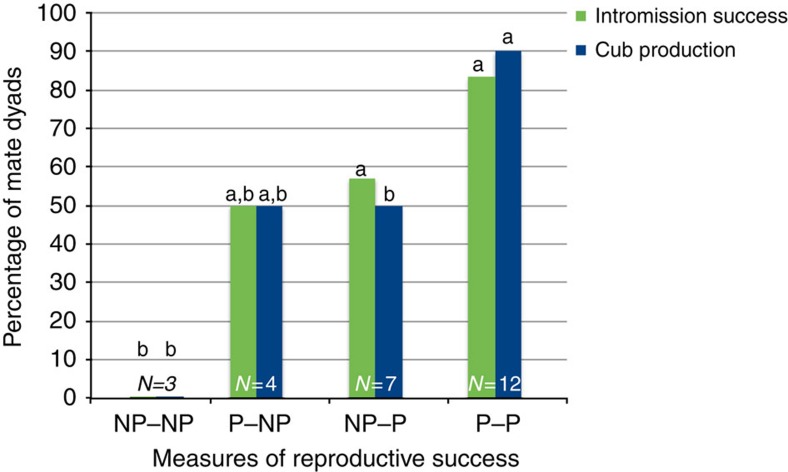
Percentage of mate dyads for different combinations of male and female preference. Male is non-preferred and female is non-preferred (NP–NP), female prefers male but male does not prefer female (P–NP), female does not prefer male but male prefers female (NP–P), and both animals prefer each other (P–P). Dark bars represent intromission success and light grey bars represent cub production. *N* indicates number of dyads total in the group. Different letters (a,b) indicates significant differences among groups on intromission success and cub production (*P*≤0.05 using a Tukey HD *post hoc* test, actual statistics presented in Table 3 and in the text).

This experiment was conducted with four treatment groups composed of the possible mate preference combinations for giant panda mate dyads: (1) both giant pandas preferred each other (P–P); (2) the female preferred the male but the male did not prefer the female (P–NP); (3) the female did not prefer the male but the male did prefer the female (NP–P); and (4) neither the female nor the male preferred each other (NP–NP). Here and throughout the text, tables and figures we designate the female preference first and the male preference second for the treatment dyads. One-way analysis of variances (ANOVAs) revealed a significant difference across the four treatments in both intromission success and cub production but not for maternal rearing of cubs (Table 3). Tukey HD *post hoc* analyses indicated that P–P and NP–P pairings had significantly more successful intromissions than NP–NP mate pairings and P–P pairings had more cubs than both NP–NP and NP–P pairings. Examination of Fig. 3 reveals that when both partners are non-preferred, no intromission or cub production occurred. When one member of the dyad was paired with a preferred partner, values of reproductive performance were intermediate. When both members of a dyad were paired with preferred partners, intromission success and cub production approached maximum reproductive performance, with 75% of pairings producing offspring. Of the remaining variables only male mass made any statistical contributions to reproductive outcomes (Table 3). Tukey HD *post hoc* analyses indicated that P–P pairings involved significantly heavier males than both NP–NP and P–NP pairings (Table 3).

## Discussion

Conservation breeding programmes represent the final step to rescue species declining toward extinction, but if they are going to be a cost-effective tool for species recovery many improvements will be needed. Our research with the giant panda indicates that better integration of behavioural research into breeding management may yield large returns on investment, even for a species that has already seen large increments of improvement from previous applications of behavioural and biological knowledge^26^,^27^,^28^. Using a strong evolutionary-ecological theoretical framework developed with animals more amenable to experimentation, we applied lessons learned regarding the value of allowing free mate choice. While expression of mate preferences are known to yield fitness benefits in other species^19^,^29^,^30^,^31^,^32^,^33^, we found surprisingly large effect sizes for mate preferences on reproductive performance for this iconic species representing conservation in general, and conservation breeding in particular. Despite considerable resources devoted to breeding giant pandas and substantive improvements in all aspects of husbandry and reproduction^28^, ours is the first study to rigorously examine the effects of mate preference in giant pandas. Although a number of other husbandry variables were incorporated in our analysis, none received the level of statistical support found for mate preferences, which were associated with much higher levels of copulation and offspring production. The only exception was evidence for increased reproductive performance in mate pairs that had previously produced a cub, however, we suggest that this finding might, in fact, be due to previous mate preferences being expressed.

Further, our study is one of few that have examined the reproductive consequences of male mating preferences and the first to do so in a conservation breeding programme. A striking finding, then, is that male mate preference was equally important for contributing to positive reproductive outcomes as was female mate preferences. And the highest reproductive performance was seen when both males and females showed mutual preference. In the past the role of male mate choice has largely been relegated to species showing sex role reversal^23^. Today our understanding is expanding, and on theoretical grounds males are predicted to show greater mate preferences when they invest heavily in competition with other males or in searching for and courting females^23^. Giant pandas conform to this prediction, investing considerable time and resources in the pursuit of a small number of females^34^ and, as we now understand, also display behavioural preferences for females that correlate with reproductive performance. This historical neglect of male mate preferences should be addressed in other species.

The failure to address behavioural preferences for mates is not limited to the giant panda programme. Despite ample recent evidence indicating fitness consequences of mate choice across many taxa^15^, beginning with Darwin's theory of sexual selection^35^, to our knowledge the effects of mate preferences on reproductive output has been examined for only one other conservation-dependent species^16^. Mate choice may result in higher fitness for a number of reasons, including higher reproductive output due to behavioural compatibility, selection for ‘good genes', or selection for genetic compatibility wherein certain allelic combinations yield higher fitness in offspring^14^,^15^, the latter including selection for optimal mean kinship^36^. Mate choice may also reasonably be expected to improve the welfare of individuals in conservation breeding programmes both for the mating adults and for the offspring through higher survival rates and increased parental care^37^. Our data do not identify mechanism of benefit for giant pandas, although increased copulation rates suggest improved behavioural compatibility and increased offspring production suggests other mechanisms such as genetic compatibility are in operation.

We acknowledge that free mate choice will not be a panacea for conservation breeding and that when incorporating it into conservation breeding programmes managers must also necessarily continue to address the overall goals of genetic population management. Mate choice may result in reduced gene diversity and founder representation if certain individuals consistently fail to be ‘preferred'^38^ and do not contribute genetically to the population. However, this is also a risk when behaviourally incompatible pairs are established on pure population genetic health parameters unless their genes are incorporated via assisted reproduction techniques. Conducting mate choice trials only among genetically suitable mates as identified by analyses to optimize outbreeding, as we have done here with giant pandas, makes it possible to have the best of both worlds^38^. Further, knowledge of behavioural mate choice mechanisms allows us to manipulate those mechanisms to encourage genetically suitable partners to mate, thus incorporating breeders into the population that might not otherwise attain genetic representation^39^.

Based on our data, incorporation of mate preferences into giant panda breeding could greatly increase reproductive rates, thereby providing an even greater surplus of giant pandas for reintroduction (Fig. 3). As China embarks on its carefully planned and long-anticipated reintroduction programme, it will need to increase the number of giant pandas produced. The number of animals released is a powerful predictor of reintroduction success^8^,^9^. Furthermore, China needs to allocate its conservation resources carefully and can ill-afford to invest in captive breeding programmes with only modest rates of return on investment.

The future of conservation breeding will not take place in a test tube; although remarkable advances have been made in human-assisted reproduction, the most cost-effective way to manage assurance populations and provide animals for reintroduction is to breed them naturally. To do that requires better understanding of natural mating behaviour. Mate choice has an important role to play in conservation because it influences population-level genetic diversity and extinction risk^40^. Unfortunately, in conservation breeding programmes, mates are traditionally selected primarily on the basis of genetic parameters to minimize loss of genetic diversity and inbreeding coefficients. Selecting breeding pairs in this manner potentially limits mate choice mechanisms, reducing population productivity and interfering with evolutionary processes supporting the genetic health of the population. Thus, our findings could have significant ramifications for captive populations of endangered species and could assist with the establishment of revised captive breeding protocols, placing behavioural compatibility on par with genetic management as a guiding principle.

## Methods

### Experimental organism

The experiments were conducted at the Bifengxia Chinese Conservation and Research Center for the Giant Panda in the Sichuan province of China. Data were collected during the breeding season (1 February–1 May) of 2012 and 2013. Giant pandas were housed in concrete walled, open-air enclosures (8 × 25 m) that contained various forms of environmental enrichment (for example, climbing platforms, water features and trees, and so on) and an indoor enclosure area (3 × 8 m). All enclosures had three barred ‘howdy' windows and a circular barred gate located on the long sides of the enclosure (eight potential interaction windows, four per side). Thus, giant pandas were able to interact through cage bars with neighbouring individuals in adjoining enclosures, but opportunities for physical contact were limited. Enclosures were arranged in a large U-shape so giant pandas could be moved freely between pens for mate pairings. In this configuration, giant pandas shared walls with two other animals, allowing for dichotomous choice tests to be performed before mate pairings, except for animals residing in the end enclosures, which only had one neighbour. All experiments were conducted during the breeding season between February and April of 2012–2013.

Giant pandas were exposed to natural light conditions. Giant pandas were fed a diet of local bamboo supplemented with bread, high-fiber biscuits, carrots and apples. Animal care and use guidelines of the American Society of Mammalogists (Animal Care and Use Committee 1998; Assurance #: A3675-01) were followed by all facility operators.

### Mate familiarity

Throughout this paper we use the term mate dyad to refer to male–female pairs introduced via a dichotomous choice test and subsequently for an attempted mating. Studbooks and veterinary records were consulted to determine if mate dyads had previously produced litters successfully^41^,^42^. As familiarity has been shown to affect mate preference^39^, we collected data on several factors that may indicate familiarity between mate dyads that were obtained from studbook and veterinary records or from accurate recording of enclosure moves throughout the year. Factors included: previous offspring production with the potential mate (binary response variable: yes=1, no=0), familiarity status during the year before breeding (number of days in adjoining enclosures), familiarity status during the month before breeding (in days), familiarity status directly before breeding (number of contiguous days animals were neighbouring each other). Familiarity status directly before breeding was highly variable ranging from 30 min to 10 days.

### Mate preference

As described above, female and male giant pandas included in mate preference trials were housed between conspecifics of the opposite sex and at least one of these neighbours was designated as a potential mate for that season as designated by the species survival plan. Mate preference behaviour was scored 1–3 days before an opposite sex conspecific was introduced to the focal animal for mating. All occurrences of the focal animal's behaviours as defined below were scored for 30 min during the active period between 0730 and 1,100 hours.

Pre-mating behaviours known to be important indicators of impending oestrus in females and sexual arousal in males were recorded: scent-marking, urination, water play, rolling, feeding activity, activity level, interest and interaction with opposite sex conspecifics, chirping, bleating, masturbation, tail up (female only), lordosis (female only), backward walking (female only), penile erection (male only) and foot scraping (male only). Also included were behaviours that may indicate negative interactions such as aggression through attempted physical attacks, lack of interest in opposite sex conspecifics, moaning, barking, growling, roaring and avoidance.

Animals were defined as preferred if the focal animal directed >60% of its total behaviours toward one particular potential mate. If the focal giant panda did not demonstrate such a preference they were excluded from the study and further analysis (*N*=5). The observations were conducted with a single-blind trial protocol, wherein observers were naive to the identity of the conspecific that would be paired with the focal giant panda.

These experimental manipulations resulted in three data sets used for analyses: (1) female focal animal mate preference trials (*N*=41); (2) male focal animal mate preference trials (*N*=40); and (3) mutual mate preference trials obtained from the above trials (*N*=26 mate dyads). For the third data set, ‘mutual mate preference', we divided mating dyads into four possible mating categories as outlined above.

### Mating procedure

Here we define mate pairings as the introduction of a specific male to a specific female for the purpose of breeding. All pairings were governed by genetic recommendations from the species survival plan; thus, some pairings were consistent with the giant panda's behavioural preference and some were not. Mating was always attempted first with the priority male according to the genetic management plan even if animals appeared indifferent or slightly aggressive toward the potential mate, but mating introductions were not attempted where excessive aggression was observed.

Female oestrus status was determined using enzyme-immunoassay for oestrogen metabolites (estrone-3-glucuronide) previously validated on urine^43^. Urine samples were collected via syringe from the enclosure floor ∼3 days a week and stored at −20 °C until analysis at the CCRCGP laboratory. During the peri-ovulatory period urine samples were collected daily. Ovulation is indicated by a>6-fold elevation of oestrogen above baseline levels, followed by a return to baseline^43^. All mating introductions were conducted during this peri-ovulatory period including the day before, the day of, and the day following presumed ovulation.

Males were introduced to female pens for mating between 0900 and 1,100 hours. Mating sessions lasted on average 15.5 min but ranged from 3 to 75 min. If either animal's behaviour was aggressive, animal care staff removed the male immediately to prevent injury or death. After a mating session, males were moved back to their enclosures and subsequently placed with a different female until all females had been mated to their recommended males. This method resulted in females being introduced to 1–4 males and having on average 4 (but as many as 9) mating opportunities each breeding season. As a fail-safe, female giant pandas are often artificially inseminated following natural breeding. If paternity was in question, the CCRCGP established the father using DNA obtained from hair samples and amplified using the polymerase chain reaction to analyze microsatellite loci after the methods of Zhang *et al.*^44^. All cubs used in this study had confirmed paternity.

### Response variables and additional independent variables

We monitored several response variables indicative of reproductive performance: whether a mating attempt failed or succeeded (that is, copulation occurred with intromission), whether cubs were produced, and whether cubs were hand raised or mother raised. In addition to familiarity and mate preference measures, we consulted veterinary and husbandry records to acquire additional independent variables that might influence reproductive performance: provenance (captive- versus wild-born), rearing history, age, morphometric measurements of size and mass, and measures of recent mating history.

### Data analyses

All statistical significance tests done in the manuscript were two sided. Although a formal power analysis was not performed, we sought to include the largest sample size available for this species. From past experience with behavioural research with this species, we determined that this sample size would be sufficient to detect a biologically meaningful result.

Before analysis variables were examined for normality, linearity and homoscedasticity. Although the distribution for some data sets deviated modestly from normality, GLMM is robust to such deviations within the explanatory variables, thus, no transformations were conducted for ease of interpretation^45^,^46^. We used a GLMM with a logit link and binomial error distribution to test whether the independent variables explained significant variance in the probability of intromission success, cub production and maternal care. Female and male identity were modelled as random effects. We ran GLMM through a step-wise exclusion method in which the least significant predictor variable (*P*≥0.05) was sequentially removed from the model until the final significant model was revealed^47^. Models were compared using penalized log likelihood scores (BIC^48^). Following standard practices for GLMM, all variables included in the final model are reported as substantive contributors to model outcome, including those that do not attain significant *P* values; this is done on theoretical grounds addressing the historical over-reliance on *P* values, specifically when using GLMM in this context^25^.

We analysed three measures of reproductive performance using GLMM in SPSS (SPSS 19.0 for Mac OS X). The first response variable was whether a mating attempt resulted in copulation (binary response variable: yes=1, no=0; GLMM with logit link function). The second response variable was whether cubs were produced (binary response variable: yes=1, no=0; model with logit link function). The third response variable was whether or not a female raised her cub or humans intervened and hand raised the cub due to maternal abandonment or incompetence (binary response variable: hand reared=1, mother reared=0; model with logit link function). The potential explanatory variables/factors that could affect the success of a mating attempt were: previous litter production with the potential mate (binary response variable: yes=1, no=0), familiarity status directly before mating sessions (in contiguous days animals were neighbouring each other), mate preference status (binary response variable: preferred=1, non-preferred=0), focal animal birth location (that is, provenance; binary response variable: captive=1, wild=0), focal animal rearing (binary response variable: mother=1, hand=0), female age (years), female mass (kilograms), female length (centimeters), female height (centimeters), male age (years), male mass (kilograms), male length (centimeters) and male height (centimeters). Some variables showed a high degree of intercorrelation. Since even small to modest intercorrelations between explanatory variables can lead to significantly different (and often erroneous conclusion) in GLMMs^49^,^50^, we have retained the variable with the most relevance to the hypothesis and most normal distribution for analysis. For example, male mass was significantly and highly correlated with male length (*r*=−0.59, *P*<0.001) and height (*r*=−0.64, *P*<0.001). Thus, male mass was the only factor used to describe male size. Likewise, female mass was significantly correlated with female length (*r*=0.22, *P*=0.05) and height (*r*=−0.29, *P*=0.03), and therefore, female mass was the only factor used to describe female size. All explanatory variables were then fitted in all possible combinations to create a list of explanatory models. We used the BIC to rank the explanatory models.

Regression analyses generally assume that all observations are independent; however, this is not the case for pairwise data, where the same individual may be involved in multiple mating attempts. Even though dyads could potentially be considered independent observations because a specific mating dyad never occurred more than once in our data set, we accounted for dyadic non-independence by including giant panda ID as a random effect in our analyses^51^,^52^. There were 11 unique male giant pandas and 27 unique female giant pandas represented in our data set. Male age ranged from 6 to 14 while female age ranged from 5 to 18.

For the purpose of graphing, we ran *χ*^2^ tests to analyze differences between preferred and non-preferred mates on intromission success, cub production and maternal care for both male and female giant pandas in R Studio (Version 0.98.981; R Studio Inc. 2009-2013; R Version 3.0.2). For the mutual mate preference data analyses we tested mating dyad reproductive performance measurements using one-way ANOVAs with a single factor using the four mating combinations as treatments (P–P, P–NP, NP–P, NP–NP) using R Studio (Version 0.98.981; R Studio Inc. 2009-2013; R Version 3.0.2). The ANOVAs that were significant or trending toward significance (*P*≤0.07) were followed by Tukey HD *post hoc* tests to examine mean differences between treatments while controlling for familywise error.

## Additional information

**How to cite this article**: Martin-Wintle, M. S. *et al.* Free mate choice enhances conservation breeding in the endangered giant panda. *Nat. Commun.* 6:10125 doi: 10.1038/ncomms10125 (2015).

## Supplementary Material

Supplementary Information Supplementary Tables 1-4

## Figures and Tables

**Table 1:** Measures of reproductive performance for mate pairings of female giant pandas.

**Variable**	Pairing type*			
	**Preferred**	**Non-preferred**	**Previous cub**	**No previous cub**
Total mate pairings	25	16	10	34
Total successful intromissions†	72.0%	31.25%	100.0%	47.1%
Cubs produced†	88.8%	40.0%	90.0%	68.8%
Mother reared cubs†	81.25%	100.0%	88.8%	72.7%
Mean number of cubs	1.4 (0.28)	1 (0.71)	1.2 (0.38)	1.5 (0.47)
Mean male age (years)	13.2 (2.63)	10.5 (2.63)	13.5 (4.08)	11.7 (2.00)
Mean female age (years)	11.1 (2.22)	10.7 (2.67)	12.7 (3.83)	10.2 (1.76)
Mean male body mass (kg)	115.8 (23.16)	112.4 (28.11)	126.6 (38.18)	111.0 (19.04)
Mean female body mass (kg)	109.7 (21.93)	112.7 (28.17)	108.9 (32.83)	110.55 (18.96)

^*^Parentheses are s.e.s.

^†^Proportion scaled to the variable above (for example, total successful intromissions out of total mate pairings).

**Table 2:** Measures of reproductive performance for mate pairings of male giant pandas.

**Variable**	Pairing type*			
	**Preferred**	**Non-preferred**	**Previous cub**	**No previous cub**
Total mate pairings	24	16	11	37
Total successful intromissions†	75.0%	31.25%	90.9%	43.2%
Cubs produced†	77.77%	60.0%	80.0%	68.75%
Mother reared cubs†	92.9%	66.66%	100.0%	72.7%
Mean number of cubs	1.4 (0.38)	1.7 (0.96)	1.3 (0.44)	1.6 (0.49)
Mean male age (years)	12.0 (2.46)	11.3 (2.83)	13.7 (4.14)	11.2 (1.83)
Mean female age (years)	11.1 (2.26)	11.1 (2.78)	13.2 (3.97)	10.6 (1.74)
Mean male body mass (kg)	124.1 (25.32)	113.3 (28.31)	127.8 (38.54)	112.6 (18.51)
Mean female body mass (kg)	110.2 (22.50)	110.7 (27.68)	110.4 (33.28)	110.26 (18.12)

^*^Parentheses are s.e.s.

^†^Proportion scaled to the variable above (for example, total successful intromissions out of total mate pairings).

**Table 3:** Components of reproductive performance by mutual mate preference in giant pandas.

**Variable**	Pairing type*				**Test**	***P***
	**P–P**	**P–NP**	**NP–P**	**NP–NP**		
Total mate pairings	12	4	7	3		
Total successful intromissions†	83.3%^a^	50.0%^a,b^	57.1%^a^	0.0%^b^	*F*_3,22_=2.97	0.05
Cubs produced†	90.0%^a^	50.0%^a,b^	50.0%^b^	0.0%^b^	*F*_3,22_=3.37	0.04
Mother reared cubs†	88.8%	100.0%	100.0%	0.0%	*F*_1,9_=0.14	0.87
Mean number of cubs	1.4 (0.48)	1	1 (0.71)	0	*F*_1,9_=0.90	0.44
Mean male age (years)	12.3 (0.82)	13.3 (0.75)	11.4 (1.13)	8.7 (0.88)	*F*_3,22_=0.90	0.45
Mean female age (years)	10.9 (1.16)	8.8 (1.43)	11 (1.69)	13.3 (0.67)	F_3,22_=0.87	0.47
Mean male body mass (kg)	123.6 (2.01)^a^	111.5 (8.83)^b^	122.3 (3.37)^a,b^	96 (4.58)^b^	*F*_3,22_=2.71	0.06
Mean female body mass (kg)	107 (6.95)	105.3 (11.05)	114.3 (3.24)	109.0 (3.51)	*F*_3,22_=0.72	0.55

NP, non-preferred; P, preferred.

Numbers or mean values (s.e.), *χ*^2^ tests, and *P* values for various traits related to litter production by females that were mated with either a P or NP male. For each mating type, the female's preference of mate (P or NP) is given first, followed by the male's preference of mate. Different superscript letters (a,b) indicate significant differences (*P*<0.05) using a Tukey HD *post hoc* test.

^*^Parentheses are s.e.s.

^†^Proportion scaled to the variable above (for example, total successful intromissions out of total mate pairings).
