# Supplementary material for: Free mate choice enhances conservation breeding in the endangered giant panda
Source: Nat Commun. 2015 Dec 15;6:10125. doi: 10.1038/ncomms10125 (PMC4682106; doi:10.1038/ncomms10125)
Supplement: Supplementary Information — Supplementary Tables 1-4 [file ncomms10125-s1.pdf]

Supplementary Table 1. Summary of a generalized linear mixed model analysis of intromission[a] for female giant panda's preferred and nonpreferred mates.

Explanatory variables	B[b]	s.e. [b]	Wald Chi-Square[b]	d.f. [b]	p[b]
Fixed effects					
Intercept	2.61	0.34	45.51	1	<0.001*
Preference	2.63	0.02	155.21	1	<0.001*
Neighbor Status (days)	-1.49	0.89	-0.17	1	0.866
Pair had cubs previously	0.65	0.23	0.03	1	<0.001*
Provenance	1.76	1.53	1.14	1	0.253
Rearing	1.22	1.59	0.77	1	0.441
Female age (years)	0.16	0.20	0.83	1	0.408
Female mass (kg)	-0.05	0.07	-0.68	1	0.500
Male age (years)	0.69	0.43	1.60	1	0.109
Male mass (kg)	0.08	0.03	3.02	1	0.003*
BIC					175.26

a. As a function of preference, neighbor status, if the pair had successfully had cubs previously, provenance, rearing, female mass and age, male mass and age, (N=41, Unique Female=27, Unique Male=11). The response variable was assumed to be binomial, given random effect for female and male identity. After a successive exclusion of the least significant terms (p≥0.05), the significance values of the final model are shown in the table. Non-significant terms are presented with the values they were removed from the model. Male and female id were treated as random effects.
b. df is degrees of freedom, B is the logistic regression coefficient, s.e. is the standard error, t denotes the t-value and p the significance level.
*denotes significant factors in final model with highest BIC

Supplementary Table 2. Summary of generalized linear mixed model of cub production for female giant pandas[a] using step-wise .

Explanatory variables	B[b]	s.e. [b]	Wald Chi-Square[b]	d.f. [b]	p[b]
Fixed effects					
Intercept	22.30	4.35	26.34	1	<0.001
Preference	4.91	1.46	11.35	1	0.001*
Neighbor Status (days)	0.24	0.23	1.10	1	0.294
Pair had cubs previously	2.197	0.94	-.349	1	0.020
Provenance	-1.92	3.39	0.32	1	0.572
Rearing	-3.74	1.60	5.48	1	0.019*
Female age (years)	-0.33	0.21	2.45	1	0.118
Female mass (kg)	0.04	0.08	0.33	1	0.568
Male age (years)	-0.46	0.18	6.93	1	0.008*
Male mass (kg)	-0.17	0.05	12.28	1	<0.001*
BIC					31.48

a. As a function of preference, neighbor status, if the pair had successfully had cubs previously, provenance, rearing, female mass and age, male mass and age, (N=41, Unique Female=27, Unique Male=11). The response variable was assumed to be binomial, given random effect for female and male identity. After a successive exclusion of the least significant terms (p≥0.05), the significance values of the final model are shown in the table. Non-significant terms are presented with the values they were removed from the model. Male and female id were treated as random effects.
b. d.f. is degrees of freedom, B is the logistic regression coefficient, s.e. is the standard error, t denotes the t-value and p the significance level.
*denotes significant factors in final model with highest BIC

Supplementary Table 3. Summary of a generalized linear mixed model of intromission for male giant pandas[a].

Explanatory variables	B[b]	s.e. [b]	Wald Chi-Squareb	d.f. [b]	p[b]
Fixed effects					
Intercept	-2.52	0.87	8.43	1	0.004
Preference	1.44	0.87	2.70	1	0.100*
Neighbor Status (days)	-0.04	0.10	0.14	1	0.712
Pair had cubs previously	2.01	1.10	3.32	1	0.069*
Provenance	-0.88	2.18	0.16	1	0.687
Female age (years)	0.60	0.14	0.17	1	0.677
Female mass (kg)	0.05	0.04	1.47	1	0.225
Male age (years)	-0.17	0.22	0.59	1	0.441
Male mass (kg)	-0.10	0.74	1.74	1	0.187
BIC					49.73

a. As a function of preference, neighbor status, if the pair had successfully had cubs previously, provenance, rearing, female mass and age, male mass and age, (N=41, Unique Female=27, Unique Male=11). The response variable was assumed to be binomial, given random effect for female and male identity. After a successive exclusion of the least significant terms (p≥0.05), the significance values of the final model are shown in the table. Non-significant terms are presented with the values they were removed from the model. Male and female id were treated as random effects.
b. d.f. is degrees of freedom, B is the logistic regression coefficient, s.e. is the standard error, t denotes the t-value and p the significance level.
*denotes significant factors in final model with highest BIC

Supplementary Table 4. Summary of a generalized linear mixed model of cub production for male giant pandas[a].

Explanatory variables	B[b]	s.e.[b]	Wald Chi-Squareb	d.f.[b]	p[b]
Fixed effects					
Intercept	23.88	9.90	5.82	1	0.016
Preference	1.64	1.08	2.32	1	0.128*
Neighbor Status (days)	0.01	0.19	0.002	1	0.964
Pair had cubs previously	0.17	1.01	0.03	1	0.867
Provenance	-3.532	1.11	10.15	1	0.001
Female age (years)	0.02	0.09	0.03	1	0.870
Female mass (kg)	0.05	0.03	2.73	1	0.980
Male age (years)	-0.58	0.16	0.14	1	0.712
Male mass (kg)	-0.20	0.08	5.90	1	0.015*
BIC					42.01

a. As a function of preference, neighbor status, if the pair had successfully had cubs previously, provenance, rearing, female mass and age, male mass and age, (N=41, Unique Female=27, Unique Male=11). The response variable was assumed to be binomial, given random effect for female and male identity. After a successive exclusion of the least significant terms (p≥0.05), the significance values of the final model are shown in the table. Non-significant terms are presented with the values they were removed from the model. Male and female id were treated as random effects.
b. d.f. is degrees of freedom, B is the logistic regression coefficient, s.e. is the standard error, t denotes the t-value and p the significance level.
*denotes significant factors in final model with highest BIC
